# Advanced Platelet Lysate Aerogels: Biomaterials for Regenerative Applications

**DOI:** 10.3390/jfb15020049

**Published:** 2024-02-19

**Authors:** Fahd Tibourtine, Thibault Canceill, Andrea Marfoglia, Philippe Lavalle, Laure Gibot, Ludovic Pilloux, Clementine Aubry, Claire Medemblik, Dominique Goudouneche, Agnès Dupret-Bories, Sophie Cazalbou

**Affiliations:** 1 CIRIMAT, Université Toulouse 3 Paul Sabatier, Toulouse INP, CNRS, Université de Toulouse, 118 Route de Narbonne, CEDEX 9, 31062 Toulouse, France; fahd.tibourtine@univ-tlse3.fr (F.T.);; 2 Département Odontologie, Faculté de Santé, Hôpitaux de Toulouse, Université Paul Sabatier, 3 Chemin des Maraichers, CEDEX 9, 31062 Toulouse, France; thibault.canceill@univ-tlse3.fr; 3 Laboratoire de Génie Chimique, Université Toulouse 3 Paul Sabatier, Toulouse INP, CNRS, Université de Toulouse, 31062 Toulouse, France; ludovic.pilloux@univ-tlse3.fr; 4 Institut National de la Santé et de la Recherche Médicale, Inserm UMR_S 1121 Biomaterials and Bioengineering, 67085 Strasbourg, France; philippe.lavalle@inserm.fr (P.L.); claire.medemblik@gmail.com (C.M.); 5 Laboratoire Softmat, Université de Toulouse, CNRS UMR 5623, Université Toulouse III—Paul Sabatier, 31062 Toulouse, France; laure.gibot@cnrs.fr; 6 ARNA, Inserm U1212, CNRS 5320, University of Bordeaux, 146 Rue Léo Saignat, CEDEX, 33076 Bordeaux, France; clementine.aubry@ubordeaux.fr; 7 Centre de Microscopie Electronique Appliquée à la Biologie, Faculté de Médecine, 133 Route de Narbonne, 31062 Toulouse, France; dominique.goudouneche@univ-tlse3.fr; 8 Department of Surgery, University Cancer Institute of Toulouse—Oncopole, 1 Avenue Irène Joliot-Curie, 31100 Toulouse, France; 9 Department of Ear, Nose and Throat Surgery, Toulouse University Hospital—Larrey Hospital, 31400 Toulouse, France

**Keywords:** human platelet lysate, supercritical carbon dioxide, aerogel, tissue repair, biomaterials, advanced therapy medicinal products (ATMPs)

## Abstract

Human platelet lysate (HPL), rich in growth factors, is increasingly recognized for its potential in tissue engineering and regenerative medicine. However, its use in liquid or gel form is constrained by limited stability and handling difficulties. This study aimed to develop dry and porous aerogels from HPL hydrogel using an environmentally friendly supercritical CO_2_-based shaping process, specifically tailored for tissue engineering applications. The aerogels produced retained their three-dimensional structure and demonstrated significant mechanical robustness and enhanced manageability. Impressively, they exhibited high water absorption capacity, absorbing 87% of their weight in water within 120 min. Furthermore, the growth factors released by these aerogels showed a sustained and favourable biological response in vitro. They maintained the cellular metabolic activity of fibroblasts (BALB-3T3) at levels akin to conventional culture conditions, even after prolonged storage, and facilitated the migration of human umbilical vein endothelial cells (HUVECs). Additionally, the aerogels themselves supported the adhesion and proliferation of murine fibroblasts (BALB-3T3). Beyond serving as excellent matrices for cell culture, these aerogels function as efficient systems for the delivery of growth factors. Their multifunctional capabilities position them as promising candidates for various tissue regeneration strategies. Importantly, the developed aerogels can be stored conveniently and are considered ready to use, enhancing their practicality and applicability in regenerative medicine.

## 1. Introduction

Platelets are circulating anucleate cell fragments in the blood that play a crucial role in the process of coagulation and the beginning of wound healing [1]. In case of injury, platelets aggregate onto the wounded vessel walls and activate to form a blood clot to prevent blood loss at the site of injury [2]. Besides their primary role in haemostasis and thrombosis, platelets participate in numerous other physiological and pathological processes, including but not limited to inflammation, wound healing, tumour metastasis, and angiogenesis, via the secretion of different bioactive molecules such as growth factors, coagulation factors, adhesion molecules, and chemokines stored initially in their alpha granules, the principal storage granule of platelets [3]. 

Recently, several studies have focused on the design of hemoderivative biomaterials based on platelet derivatives such as platelet-rich plasma (PRP) and platelet lysate (PL) as they have great potential for use in regenerative therapy as autologous sources of growth factors [4]. They can be obtained using simple and cost-effective procedures and used in personalized medicine approaches. The autologous application of endogenous growth factors considerably reduces the risks of disease transmission and simultaneously allows for the induction of the “wound healing cascade” in a physiological manner.

Human platelet lysate (HPL) is derived from human platelet concentrates through a mechanical or chemical disruption of platelet membranes [5].Growth factors (GFs) such as insulin-like growth factor-1 (IGF-I), platelet-derived growth factor (PDGF), vascular endothelial growth factor (VEGF), fibroblast growth factor (FGF), epidermal growth factor (EGF), and platelet-derived epidermal growth factor (PDEGF) have been identified in HPL [6]. These growth factors play crucial roles in different stages of wound healing, such as chemotaxis [7], angiogenesis [8], cell proliferation, and differentiation [9,10].

Previous studies have shown that it can be applied as an effective source of growth factors for tissue regeneration, such as for tendon [11], bone [12], cartilage [13], skin [14], and other organs [15]. It has also been demonstrated that HPL is more suitable for cell culture as a medium supplement compared to other animal-derived products such as foetal bovine serum [5].

In clinical applications, HPL is often applied externally to the target organ (e.g., cornea, diabetic foot ulcer…) [16], added to an implanted material [17] or injected directly into the lesion [18]. It is used either alone as a source of growth factors [19] or as a scaffold for the simultaneous delivery of cells (e.g., mesenchymal stem cell) [20]. Indeed, once associated with calcium, the HPL coagulates and thus forms a hydrogel. However, the hydrogels obtained present low mechanical properties, which result in a lack of rigidity and a tendency to adhere to surfaces, making them very challenging to handle [21]. To overcome these limitations, several studies have pursued innovative strategies, such as incorporating additional polymers (collagen, chitosan) [22]. Furthermore, the utilization of gelatine methacryloyl (GelMA), which becomes photocrosslinkable following the addition of a photo initiator, has been reported [23]. Moreover, their high-water content limits their storage duration [24], thereby adding an additional constraint to their use.

To overcome these limitations, we propose in this article the design of a dry and porous biomaterial with improved handling properties and stability. This material, presented in the form of an aerogel, is enriched with HPL and is obtained from HPL hydrogel dried through a process involving the use of supercritical carbon dioxide (scCO_2_). ScCO_2_ is a known method for preparing aerogels. It offers several advantages such as availability, non-toxicity, non-flammability, and low cost. It also confers the ability to regulate pore size and produce monolithic and solvent-free aerogels [25].

The use of aerogels in the biomedical domain has witnessed substantial advancement due to their numerous notable benefits. These include their capacity to retain a porous structure, exhibit superior mechanical strength, and maintain long-term stability. Furthermore, biopolymer-based aerogels are distinguished for their outstanding biocompatibility and biodegradability, and their potential for serving as drug delivery systems [26]. 

The development of human-derived aerogels offers a range of significant clinical advantages, enhancing their relevance for applications in regenerative medicine. Their ability to facilitate enhanced tissue regeneration stems primarily from the presence of multiple bioactive molecules within these materials, creating an environment conducive to cellular regeneration and tissue restoration. Importantly, their use also reduces the risk of disease transmission compared to animal-derived biomaterials. Their dry form confers a substantial advantage in a clinical setting, facilitating their handling, long-term storage, and immediate availability. This feature, combined with their capacity to induce a positive biological response, opens promising prospects for versatile clinical applications, including the regeneration of tissues such as skin, bone, and cartilage. These aerogels stand out for their ability to deliver growth factors, significantly enhancing the regeneration process.

## 2. Materials and Methods

### 2.1. Aerogel Preparation 

#### 2.1.1. Synthesis of Human Platelet Lysate Hydrogel

The platelet lysate hydrogel was prepared by combining human platelet lysate (HPL) (Human PL100^®^, MacoPharma, Tourcoing, France) with the following components: sodium chloride (NaCl) (Fisher scientific, Loughborough, UK) at a concentration of 0.9% (*w*/*v*), tranexamic acid at 0.1 g/mL (Acros Organics, Geel, Belgium), and anhydrous calcium chloride (CaCl_2_) at 10% (*w*/*v*) (Fisher Scientific, Illkirch, France). The HPL solution accounted for 68.7% (*v*/*v*) of the total volume of the preparation, while CaCl_2_ constituted 2.5% (*v*/*v*), NaCl 28.6% (*v*/*v*), and tranexamic acid 0.2% (*v*/*v*). Precise volumes of each constituent were calculated to achieve a final volume of 2 mL for the solution. The mixture was incubated at 37 °C for 30 min in cylindrical containers, which enabled the formation of hydrogel cylinders (approximately 1.8 cm in diameter and 1cm in height). 

#### 2.1.2. Water-Solvent Exchange of Hydrogel

**Figure 1:**
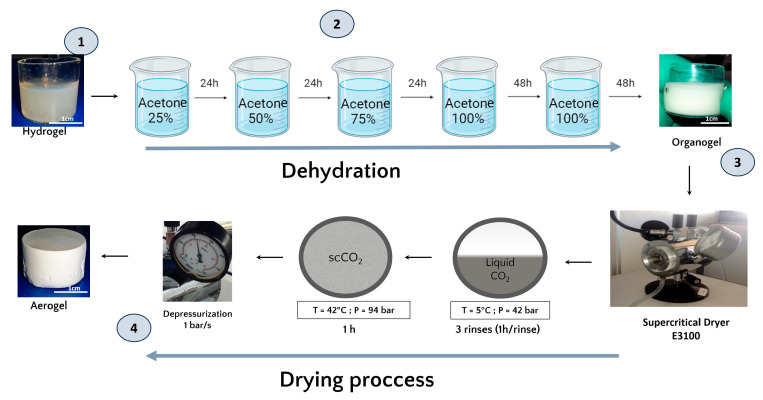
(1) The HPL hydrogel precursor. (2) Schematic representation of the acetone bath steps involved in the formation of the organogel and the subsequent dehydration and substitution of the aqueous phase with an organic phase miscible in liquid CO_2_. (3) Schematic illustration of the supercritical CO_2_ drying process. (4) The final product, the platelet lysate aerogel.

Once formed, and prior to the drying process, the hydrogels were dehydrated in successive baths of 24 h in increasing concentrations of acetone (25%, 50%, 75%, and 100%). The volume (500 mL) of acetone used was largely sufficient in volume to ensure that the water released by the hydrogel did not significantly modify the acetone concentration of the exchange solution. A total of 48 h was added to the 100% acetone baths to guarantee that the acetone water exchange was complete along with the obtention of an organogel (Figure 1) [27].

#### 2.1.3. Aerogel Production by Supercritical CO_2_ Drying 

The organogels were placed in the supercritical CO_2_ chamber (E3100 Critical Point Dryer, Quorum Technologies Ltd., Lewes, UK) and once the chamber was closed, it was filled halfway with liquid CO_2_ at 5 °C to reach a pressure of 42 bar for one hour to ensure acetone–liquid CO_2_ exchange. 

After one hour of incubation, a depressurization was performed to remove the liquid CO_2_ mixed with acetone and replaced it with fresh liquid CO_2_. This process was repeated 3 times. Then, the system was brought to the supercritical state by filling the chamber halfway with liquid CO_2_ followed by an increase in temperature to 42 °C, which increased the pressure to 94 bar and reached the supercritical phase. These conditions were maintained for one hour. Finally, the top valve of the dryer was opened for one hour at a rate of 1 bar/sec to ensure a smooth release of the CO_2_. In the end, the dryer was opened and the aerogels were collected.

### 2.2. Characterization of the Aerogels

Different characterizations were performed in order to evaluate the effect of the shaping process involving scCO_2_ on the physicochemical and bioactive properties of HPL aerogels. A comparison between HPL aerogels and HPL hydrogels, fresh or dried, at 37 °C for 72 h in a thermostatic oven was performed. The details of the techniques and parameters used are as follows.

#### 2.2.1. Scanning Electron Microscopy

The morphology and microstructure of the aerogels were compared to fresh HPL hydrogels and oven-dried HPL hydrogels using scanning electron microscopy (SEM), (Quanta 250 FEG FEI, Thermo Scientific, Waltham, MA, USA). 

The HPL hydrogels were formed as described above, then fixed overnight in 2% glutaraldehyde. After fixation, samples were dehydrated with ethanol gradient and treated with hexamethyldisilazane (HDMS) [28]. 

All samples were carefully cut into small fragments using a scalpel, ensuring their structure remained intact. They were then coated with a 6 nm layer of platinum. Observations were conducted in the secondary electron emission mode using a high voltage of 5 kV.

#### 2.2.2. Mercury Porosimetry

The porosity of the aerogels was measured using a mercury intrusion porosimeter (AutoPore III, Micromeritics Instruments Inc., Norcross, GA, USA), which allows the detection of pores in the range 360 μm to 4 nm. The pore size distribution was calculated as the differential mercury intrusion volume plotted versus the pore size. The total percentage porosity was calculated by Equation (1):

(1) $$P_{\mathrm{tot}}=d_{\mathrm{app}}\times VH_{g}\times 100\;[\%]$$

where *P*_tot_ is the percentage of total porosity, *d*_app_ is the apparent density of the scaffold, and *V*H_g_ is the total mercury intrusion volume per gram of specimen analysed. Cylinder samples were carefully divided into four equal segments using a scalpel, ensuring to maintain their structural integrity. Subsequently, one of these quarters was randomly selected for measurement. Each value was derived from three parallel measurements taken from different aerogels (*n* = 3).

#### 2.2.3. Texture Profile Analysis (TPA)

HPL hydrogels and HPL aerogels (cylindrical samples: 9 mm height, 16 mm diameter) were tested under a double compression using a TA. XT Plus Texture Analyser, (Texture Technologies, Hamilton, MA, USA) equipped with a 6 mm diameter cylinder probe and a 5 kg load cell. The samples were placed on the base plate of the texture analyser and compressed at a speed of 0.5 mm/s to a deformation of 50%. The prop was then withdrawn from the sample at the speed of 0.5 mm/s. The hardness and adhesiveness were measured for each sample (*n* = 5) using Texture Exponent 32 software (Texture Exponent 32 4.0, Stable Micro Systems Ltd., Godalming, UK).

#### 2.2.4. Hydration Test 

The cylindrical aerogel samples, each measuring 9 mm in height and 16 mm in diameter, were accurately weighed and then immersed in a phosphate-buffered saline solution (PBS) adjusted to pH 7.4. This immersion was carried out at a controlled temperature of 37 °C to closely simulate physiological conditions.

Hydration measurements were conducted at various time intervals, specifically at 0, 5, 10, 15, 20, 25, 30, 35, 60, 120, 180, 240, 300, and 360 min. After each specified time interval, the samples were meticulously removed, any excess surface liquid was delicately blotted off, and the scaffolds were then accurately reweighed. The hydration ratio of was defined as the ratio of the wet sample weight (*W*) to the initial dry sample weight (*W*_0_) (see Equation (2)):

(2) $$\mathrm{Hydration}\;\mathrm{Ratio}=w∕w_{0}\;[-]$$

The water content was investigated as the amount of liquid absorbed by the hydration, as expressed in Equation (3), where (w) is the wet sample weight and ($w_{0}$) is the dry sample weight:

(3) $$\mathrm{Percentage}\;\mathrm{of}\;\mathrm{water}\;\mathrm{content}=\frac{w-w_{0}}{w_{0}}\times 100\;[\%]$$

Each value was calculated from three parallel measurements (*n* = 3).

#### 2.2.5. Water Drop Contact Angle 

To investigate the hydrophobic/hydrophilic properties, contact angle measurements were carried out using the Drop Shape Analyser DSA30S (KRÜSS GmbH, Hamburg, Germany). A calibrated 2 µL droplet of PBS solution, adjusted to pH 7.4 and maintained at a temperature of 25 °C, was dispensed onto the surface of the samples (cylindrical samples: 9 mm height, 16 mm diameter) using a fine needle. The contact angle, formed between the droplet and the surface of samples, was subsequently measured from the captured images using the KRÜSS advance software. This procedure was replicated three times, each time on a different sample. The behaviour of the droplet was assessed continuously until its complete absorption.

#### 2.2.6. Fourier Transform Infrared (FTIR) Spectroscopy

Attenuated total reflection Fourier transform infrared (ATR-FTIR) chemical analysis of HPL aerogels and liquid HPL was conducted at room temperature for the qualitative determination of bonds presented in samples. 

For each spectrum, 64 scans between 400 and 4000 cm^−1^ were recorded, with a resolution of 4 cm^−1^ (Nicolet 5700, Thermo Electron, Waltham, MA, USA).

#### 2.2.7. Quantification and Kinetic Release of Total Protein

The total protein release from HPL aerogels and HPL hydrogels was quantified using the Pierce BCA Protein Assay Kit (Thermo Scientific, Waltham, MA, USA), adhering to the microplate assay protocol provided. Cylindrical aerogels (9 mm height, 16 mm diameter) were immersed in 5 mL of PBS (37 °C, pH 7.4). Aliquots of 500 µL were sampled at specific time points, including 0, 1, 2, 4, 24, 28, 44, 48, 52, 72, and 168 h. An equal volume of fresh PBS was added to the suspension to replace the collected samples, which were stored at −20 °C until analysis. To assess the amount of total protein in the HPL stock solution, HPL was diluted at 1:20 in PBS to achieve a concentration range corresponding to the standards. Standards were prepared according to the manufacturer’s protocol, and total protein was measured via absorbance at 560 nm using multi-well reader (CLARIOstar Plus, BMG Labtech, Germany). Data are presented as mean ± standard deviation for independent samples at each time point (*n* = 3).

#### 2.2.8. Quantification and Kinetic Release of VEGF by Enzyme Linked Immuno-Sorbent Assay (ELISA) 

The release of VEGF from HPL aerogels was determined by enzyme-linked immunosorbent assay (Human VEGF Pre-Coated ELISA Kit, Biogems, Westlake Village, CA, USA), following the supplier’s protocol. The optical density was read at 450 nm using a multi-well plate reader.

The aerogels (cylindrical samples: 9 mm height, 16 mm diameter) were immersed in 2 mL of PBS at 37 °C and pH 7,4 at predetermined time points (2, 4, 8, 24, 48, and 120 h); aliquots of 150 µL were collected and stored at −20 °C. The analysis was performed in triplicate (*n* = 3).

### 2.3. In Vitro Biological Studies 

#### 2.3.1. Metabolic Activity Measurement by MTT Assay 

To assess the integrity and functionality of growth factors released by HPL, the viability of BALB-3T3 fibroblast cells exposed to medium conditioned by the aerogels was evaluated using the MTT assay method [29]. The principle of the assay is as follows: BALB-3T3 fibroblast cells were seeded at a density of 9000 cells per well in a 96-well plate, with 100 µL of Dulbecco’s Modified Eagle Medium (HyClone™ DMEM, Cytiva, Washington, DC, USA) supplemented with 10% foetal bovine serum (FBS) (HyClone™), 100 U/mL penicillin, and 100 mg/mL streptomycin (Sigma-Aldrich Chemical Co., Saint-Quentin-Fallavier, France). After incubating for 24 h, the culture medium was replaced with 100 µL of extract medium obtained from the aerogels. This extract medium was prepared by incubating aerogels (2 mm height, 16 mm diameter) in DMEM without FBS for 24 h. The volume of DMEM used for incubating the aerogels was calculated to ensure the medium was supplemented with 10% HPL. For instance, if 1 mL of HPL was used to prepare the aerogel, it would be incubated in 10 mL of DMEM. The cells were then treated with this conditioned medium, which had been in contact with the aerogels, for a further 24 h. 

As a positive control, DMEM medium supplemented with 10% FBS was utilized, while 20% dimethylsulfoxide (DMSO) served as the negative control. Additionally, DMEM alone without FBS was employed as a control to evaluate the effect of growth factors.

Following 24 h of incubation with the extract medium and the control groups, the medium was removed and replaced with 100 µL of MTT solution (1 mg/mL final concentration) prepared in DMEM medium. The plates were subsequently incubated at 37 °C for 2 h in a humidified atmosphere containing 5% CO_2_. The MTT solution and formazan crystals were then dissolved in 100 µL of DMSO.

The optical density was measured at 570 nm using a multi-well reader, and the results were expressed as percent viability. The positive control (DMEM with 10% FBS) was considered to represent 100% viability, and thus, the viability of treated cells was calculated as a percentage relative to this control. The analysis was performed in six replicates (*n* = 6).

#### 2.3.2. Endothelial Cell Migration Assay 

To evaluate the pro-migratory potential of the products released by the different kinds of aerogels, a cell migration assay (scratch test) was conducted. For this purpose, early in the morning, 70,000 primary human endothelial cells (GFP+-HUVECs) were seeded in 96-well plates (Imagelock, Sartorius, Germany) within 100 µL of EGM-2 cell culture medium (Endothelial Cell Growth Basal Medium-2, Lonza). This seeding density allows us to obtain a confluent monolayer when cells adhere. To obtain calibrated scratch (750 µm width) in each well of a 96-well plate, Wound Maker device (Sartorius, Germany) was used on the evening of the seeding day. Supernatants were immediately removed to discard floating wounded cells. Cells were then incubated with 100 µL of conditioned media or control media (non-conditioned). The conditioned media were prepared by incubating the aerogels in complete EGM-2 for 24 h at 37 °C. The volume of medium per aerogel and the incubation period (24 h) was similar as for the MTT assay described above. Then, plates were placed in Videomicroscope Incucyte^®^ S3 (Sartorius, Germany), equipped with scratch wound analysis module. Wound closure was monitored by taking 10× pictures every hour for 24 h. Pictures were analysed using the Incucyte S3 associated software. Results are expressed as wound density, meaning cell confluence measurement within the initial margins of the wound. The analysis was performed in six replicates (*n* = 6).

#### 2.3.3. Cell Proliferation Measurement

BALB-3T3 fibroblast cells were seeded on HPL gels (2 mm height, 16 mm diameter) at a density of 200,000 cells in 2 mL. Cell proliferation was analysed at days 1, 7, 10, 20, and 30. At each time point, the metabolic activity of viable and attached cells was assessed using the Alamar Blue assay (Alamar Blue kit, Invitrogen, Waltham, MA, USA). In brief, following each incubation period, the culture medium was removed, and the samples were washed twice with Dulbecco’s phosphate-buffered saline solution (DPBS, Gibco, New York, NY, USA). The samples were then transferred to a new 24-well plate. Subsequently, 1 mL of complete DMEM supplemented with 10% FBS and 5% resazurin solution was added to each well. The cells were incubated for 2 h in a 5% CO_2_ atmosphere at 37 °C. The supernatants were then transferred to a black 96-well plate, and fluorescence was measured using a microplate reader with an excitation wavelength of 560 nm and an emission wavelength of 590 nm. This analysis was conducted in four replicates (*n* = 4).

#### 2.3.4. Scanning Electron Microscopy Approach for Cellular Observation of HPL Aerogels

BALB-3T3 fibroblast cell morphology was assessed using SEM, following the method previously described [30]. This involved rinsing the samples, similar to those used for cell proliferation assays, with a 0.125 mM cacodylate solution. Subsequently, they were fixed with 4% glutaraldehyde in a 50 mM cacodylate buffer (Thermo Scientific Chemicals, Waltham, MA, USA) for 2.5 h at room temperature. The samples then underwent dehydration through a graded series of ethanol concentrations, followed by treatment with hexamethyldisilazane (HDMS) for 30 min.

Post-dehydration, the samples were coated with a 6 nm layer of platinum using an SI50B Sputter Coater (Edwards, UK). Observations were conducted using a Quanta 250 FEG SEM (FEI, Thermo Scientific, Waltham, MA, USA), with all images captured in the secondary electron emission mode at a high voltage of 5 kV.

### 2.4. Statistical Analysis

The experiments were conducted a minimum of three times, and the results are presented as mean ± standard deviation (SD). Statistical analyses were performed using GraphPad Prism 8 software (GraphPad Software, Inc., La Jolla, CA, USA). To assess differences between groups, an unpaired t-test with Welch’s correction was employed, with statistical significance set at *p* < 0.05. Additionally, ordinary one-way analysis of variance (ANOVA) was conducted, followed by Dunnett’s multiple comparisons test, with a significance threshold of *p* < 0.05. These specific statistical tests were chosen to rigorously evaluate the obtained data and identify significant differences among experimental group.

## 3. Results and Discussion

### 3.1. HPL Aerogel Conception 

HPL, rich in bioactive molecules, including coagulation factors such as fibrinogen, thrombin, and factor XII, serves as a foundation for developing our hydrogels. The incorporation of calcium ions via CaCl_2_ addition initiates a three-dimensional fibrin network formation within the hydrogels [31].

As water is immiscible with scCO_2,_ before proceeding with supercritical CO_2_ drying, an essential step involves substituting the initially present water with acetone. To ensure structural integrity, we gradually increased the acetone concentration in incremental steps of 25% (*v*/*v*) during the substitution process [32]. Final immersion in 100% acetone was repeated to guarantee complete solvent exchange.

Supercritical CO_2_ drying allows for the production of dry HPL aerogels. The obtained aerogels exhibited stable shapes, and their dimensions were similar to those of the hydrogels, indicating no major structural collapse. This structural preservation was macroscopically observed and is depicted in Figure 1. 

**Figure 2:**
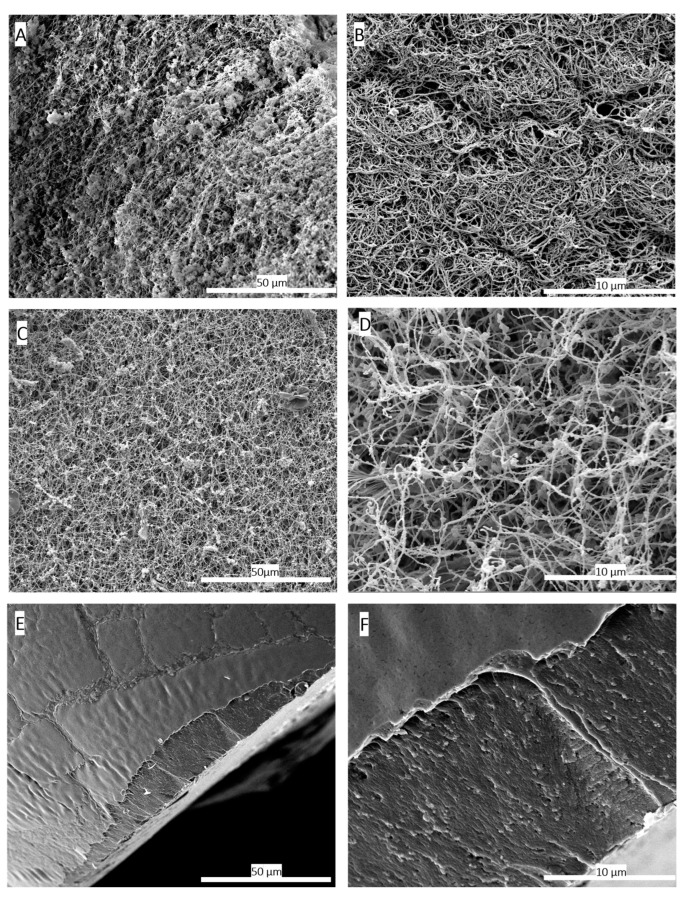
Scanning electron microscopy (SEM) images comparing the 3D structure of fibrin networks in platelet lysate hydrogels (**A**,**B**) and aerogels (**C**,**D**), along with hydrogels dried conventionally in an oven at 37 °C (**E**,**F**). (**A**) SEM image of the hydrogel at a magnification of 1000×. (**B**) SEM image of the hydrogel at a magnification of 5000×, illustrating the formed fibrin network. (**C**) SEM image of the HPL aerogel at 1000× magnification. (**D**) SEM image of the HPL aerogel at 5000× magnification, confirming the preservation of the fibrin network’s structural integrity through the supercritical CO_2_-based shaping process. (**E**) SEM image of the hydrogel dried in an oven at 37 °C at a magnification of 1000×. (**F**) SEM images of hydrogels dried in an oven at 37 °C at a magnification of 5000×. These images highlight the adverse effects of conventional drying on the three-dimensional fibrin network structure, resulting in a flattened and non-porous fibrin structure. These findings provide conclusive evidence of the destructive impact of oven drying at 37 °C on the three-dimensional fibrin network, resulting in a flattened and non-porous structure.

Scanning electron microscopy (SEM) images were utilized to compare the three-dimensional network structure between the hydrogel and aerogel obtained after scCO_2_ drying. The results of this analysis, presented in Figure 2B,D, demonstrate the preservation of the three-dimensional fibrin network. The network fibres exhibited a rough, non-smooth appearance compared to purified fibrin fibres [33]. This roughness is potentially attributed to other HPL components, especially proteins that attach to the network. Previous studies have shown that certain growth factors such as β-FGF and VEGF have an affinity for fibrin network [34].

ScCO_2_ drying is considered the gentlest method for shaping aerogels, as it prevents shrinkage or collapse of three-dimensional network of gels [35]. In contrast, conventional drying methods at room temperature or in ovens can cause structural collapse due to capillary forces [36]. This is clearly evident when comparing SEM images of HPL hydrogels dried in an oven at 37 °C with HPL aerogels obtained through scCO_2_ drying. In the case of aerogels, the three-dimensional fibrin network structure is preserved, whereas the hydrogel dried in the oven exhibits complete collapse of the three-dimensional network and pore shrinkage, as presented in Figure 2E,F, due to capillary forces [37]. The SEM observations are confirmed by the porosity measurements carried out by the mercury porosimeter on the two types of materials (Table 1). 

According to the International Union of Pure and Applied Chemistry (IUPAC), aerogels are officially defined as “microporous solids in which the dispersed phase is a gas” [38]. These materials are renowned for their exceptional properties, characterized by ultra-low density (approximately 0.003 to 0.5 g/cm³), high porosity (80% to 99.8%), high specific surface area (500 to 1200 m^2^/g), and being ultralightweight [39]. Remarkably, our fabricated aerogels exhibit exceptional porosity, averaging at approximately 93.9% (Table 1), and display a consistent density of approximately 0.09806 cm³/g, aligning closely with the defining characteristics of aerogels as established by IUPAC.

### 3.2. Mechanical Propreties Comparaison 

**Figure 3:**
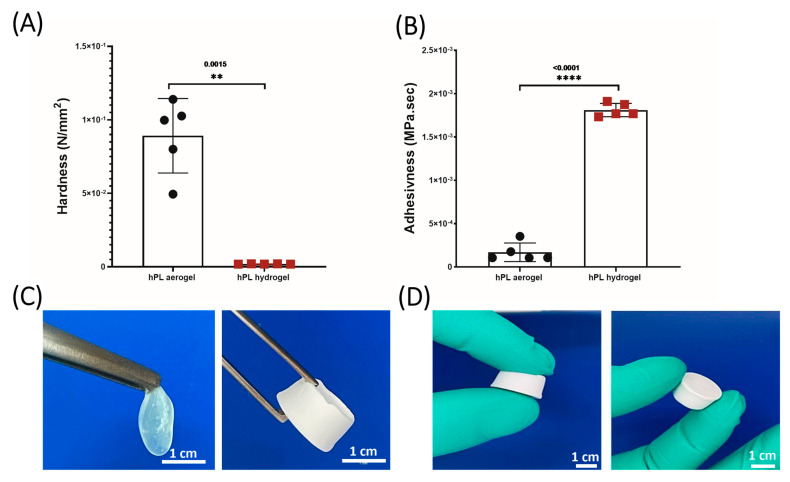
(**A**) Hardness measurements comparing hydrogels and aerogels. (**B**) Adhesiveness measurements between hydrogels and aerogels. (**C**) Macroscopic mechanical properties of hydrogels and aerogels. (**D**) Aerogel manipulability. Data represent the mean of 5 replicates, and statistical significance was assessed using unpaired t-test with Welch’s correction for *p* < 0.05 (** *p* < 0.01, **** *p* < 0.0001) (*n* = 5).

The texture profile analysis (TPA) applied to HPL hydrogels and aerogels enabled a precise evaluation of their mechanical properties and a detailed comparison of their robustness. Measurements of parameters such as hardness and adhesiveness, as presented in Figure 3, highlighted significant disparities. Hardness, defined as the maximum force required for sample compression, is derived from the peak force observed during the first compression cycle. Adhesiveness, on the other hand, assesses the material’s tendency to adhere to the contact surface with the piston and is evaluated by calculating the area under the negative curve between the two compression cycles [40]. The results clearly demonstrate that the aerogels exhibit greater hardness compared to the hydrogels, while being less adhesive, thereby significantly enhancing their manipulability, as illustrated in Figure 3C,D.

HPL hydrogels being highly hydrated, offer flexibility and limited mechanical rigidity, making them easily malleable but challenging to handle. It is established that water acts as a plasticizer for proteins, altering the interactions between protein chains and their environment, which can influence their conformation, stability, and mechanical properties [41]. For instance, in fibrin, water may reduce intermolecular interactions between fibres, thereby increasing the matrix’s flexibility. The dehydration process we employed, involving successive acetone baths followed by supercritical CO_2_ drying, reduces this flexibility, imparting rigidity without structural collapse. In contrast, the aerogels, devoid of water, solidify their structure, significantly reducing adhesiveness and thus facilitating their handling. They are therefore ideally suited for applications requiring structural stability. The developed aerogels exhibit significantly higher mechanical stability compared to hydrogels and are capable of maintaining their structure for several months, whereas hydrogels undergo complete structural collapse within a few hours due to capillary forces.

### 3.3. Hydrophobic–Hydrophilic Characteristics and Absorption Properties of HPL Aerogels

The absorption properties of aerogels are of great interest due to their ability, once implanted, to interact with surrounding fluids, facilitating the integration of vital components and key players in the tissue regeneration process, such as growth factors and cells. 

**Figure 4:**
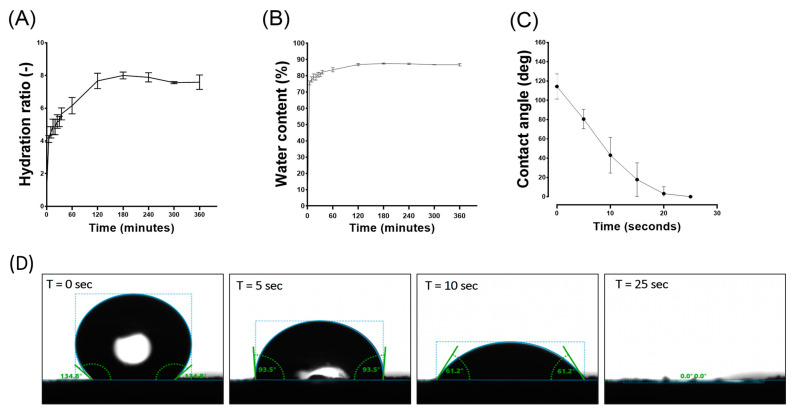
Comprehensive analysis of HPL aerogel behaviour in contact with liquid (PBS), including: (**A**) the swelling ratio, (**B**) water content, (**C**) contact angle, and (**D**) visual representation of droplet interaction. These results contribute to the understanding of the dynamic response and surface properties of HPL aerogels in a liquid environment. Error bars represent standard deviation (*n* = 3).

When placed in PBS, HPL aerogels exhibit a remarkable capacity for rapid fluid absorption. Within approximately 120 min, these aerogels attain a steady state in their swelling behaviour as illustrated in Figure 4A. Consequently, in this relatively short period, the material absorbs an impressive 87% of its own weight in PBS as shown in Figure 4B. Notably, while achieving this rapid absorption rate, the structural integrity of the samples remains unaffected, and upon rehydration, they regain an appearance akin to that of the hydrogel before drying.

Contact angle studies were conducted to analyse the hydrophobicity and hydrophilicity properties of the surface. Immediately after the deposition of the PBS droplet, an initial average contact angle of approximately 115 degrees was observed in the first few seconds, indicating a hydrophobic behaviour. The contact angle rapidly decreased until reaching 0 degrees after 25 s, indicating a hydrophilic behaviour (Figure 4C,D). This rapid absorption suggests a highly interesting haemostatic property which is crucial for the wound healing process [42].

Platelet lysate encompasses a diverse array of biological compounds, each exhibiting unique hydrophilic or hydrophobic traits. The hydrophobicity initially observed in contact angle assessments is likely attributable to the ionic interactions occurring at the surface between lipids, glycoproteins, and the established fibrin network. This interaction facilitates the formation of a surface coating that endows the material with hydrophobic properties [22]. Although this hydrophobicity is present initially, it does not hinder the rapid and significant absorption of the fluids (within seconds), which is associated with an appropriate hydrophilicity for this type of material. In addition to being influenced by the fabricated porous nature, the hydrophobicity/hydrophilicity of aerogels also depends on the other hydrophilic constituents present. Proteins, which are an essential part of platelet lysate, are composed of amino acids. Each amino acid possesses its own characteristics in terms of polarity and charge, and thus its own hydrophilicity/hydrophobicity, which explains the coexistence of both phenomena.

### 3.4. Chemical Strcture Analysis (FTIR) 

**Figure 5:**
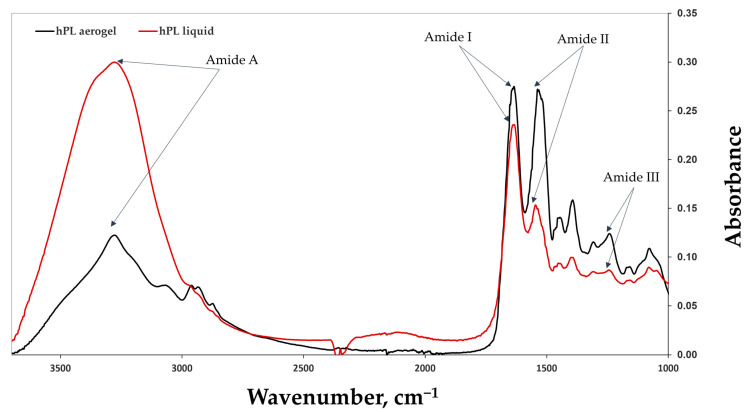
The FTIR spectra of liquid HPL (in red) and HPL aerogels (in black), highlighting the characteristic peaks of amides I, II, III, and A. The comparison between the FTIR spectra of liquid HPL and HPL aerogels allows for the assessment of any structural changes or preservation of molecular characteristics induced by the aerogel formation process. The results provide valuable insights into the chemical composition and structural integrity of HPL aerogels.

FTIR-ATR (Attenuated Total Reflectance) spectroscopy was employed to acquire spectra of human platelet lysate (HPL) aerogels and liquid HPL (Figure 5). Characteristic peaks of amide I (1633 cm^−1^/1634 cm^−1^), corresponding to C=O stretching, amide II (1546 cm^−1^/1537 cm^−1^), associated with N-H bending and C-N stretching bands, and amide III (1243 cm^−1^/1242 cm^−1^) were observed. Peaks around 1397 cm^−1^/1393 cm^−1^, characteristic of platelet lysate [22], are attributed to its various constituents. This indicates that the chemical structure is potentially unaffected by the supercritical CO_2_ drying process and is likely preserved.

In the case of aerogels, an increase in the peaks of amide II was noted. This can be explained by the emergence of C-N groups (amide II). This phenomenon may be attributed to protein–protein interactions such as fibrin network formation (fibrinogen polymerization) and interactions among other proteins in the platelet lysate, either with each other or with the formed fibrin network [43].

### 3.5. Release Kinetics of Total Proteins and VEGF from HPL Aerogels

**Figure 6:**
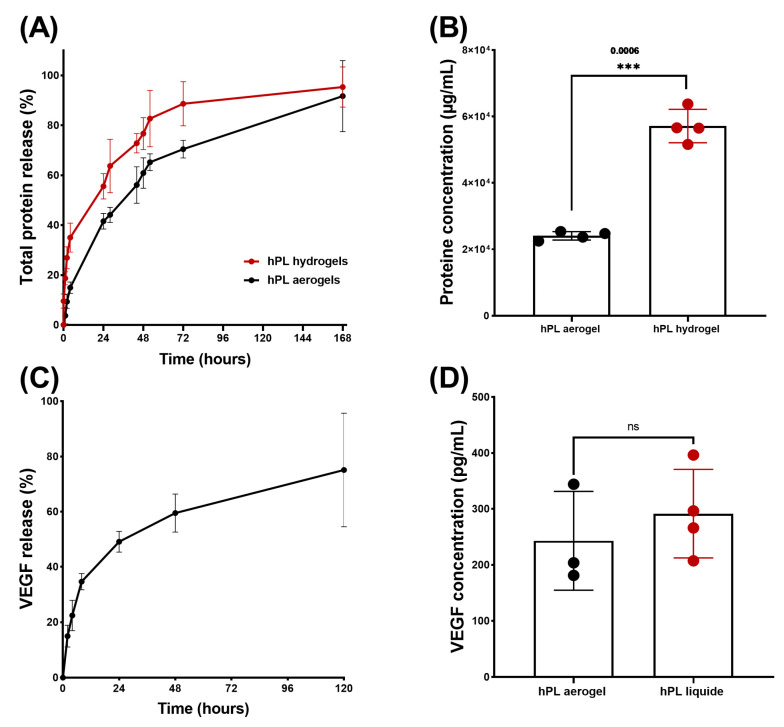
Results of release studies, quantifying total protein release and VEGF release. (**A**) Comparison of release kinetics, presented as percentages, between HPL aerogels (in black) and HPL hydrogels (in red). (**B**) Comparison of released protein concentrations between HPL aerogels and HPL hydrogels at 168 h. (**C**) Release kinetics of VEGF from HPL aerogels and HPL liquid. (**D**) Comparison of VEGF concentrations in HPL aerogels at 120 h and liquid HPL. Statistical significance assessed via unpaired *t*-test with Welch’s correction for *p* < 0.05 (*** *p* < 0.001, ns > 0.05). Error bars represent standard deviation (*n* = 3).

The cumulative release of total proteins is depicted in Figure 6A, illustrating the release profiles of proteins from both aerogels and hydrogels over time. Notably, the protein release profile from aerogels exhibited a sustained release pattern compared to hydrogels throughout the entire duration of testing. Specifically, hydrogels released 50% of the total proteins within 23 h, while aerogels extended this release to 44 h.

However, both release profiles exhibited a typical sustained release pattern which begins with: (i) rapid and high initial protein release during the first 4 h, potentially due to freely unbound proteins that can be easily removed from sample surfaces; (ii) a phase during which the free proteins contained inside the material are released by diffusion within the porous network (between 24 and 72 h); and finally, (iii) a slower and prolonged release of up to 168 h, which may be associated with the progressive degradation of the material. The release of total proteins therefore depends on several factors, such as the size of the sample and, in particular, its external surface area in contact with the liquid (first phase of release), the porosity of the material (second phase of release), and the speed of degradation of the network of fibrin (third phase of release).

Thus, the total proteins present in aerogels have a slower release speed than that observed for hydrogels due in part to the time necessary to hydrate the aerogel, deploy the fibrin fibres, and reduce the strength of the bonds created during the condensation of the growth factors on the fibrous networks.

Even though the cumulative percentage of total protein release occurs more gradually in the aerogels, which is relevant for clinical applications, it is important to note that the results obtained from protein quantification showed significantly lower protein content in the aerogels at 168 h (Figure 6B). This could be attributed to a potential protein loss during the shaping process such as the acetone baths or rinsing with liquid CO_2_ [26].

Platelet lysate is a rich source of growth factors and cytokines that play an effective role in wound healing and the recruitment of cells involved in tissues repair [44]. Among the many growth factors contained in platelet lysate, it has been shown that VEGF release plays a crucial role in angiogenesis, contributing to the formation of new blood vessels, restoring vascularization, and supporting tissue repair. It has also been demonstrated that VEGF is involved in cell recruitment and can increase vascular permeability, facilitating the passage and migration of immune blood cells that participate in the inflammatory response and healing process [45]. 

To confirm that the growth factors are still in aerogels, a release kinetics study of VEGF was conducted using an ELISA test (Figure 6C). The kinetic profile of VEGF release exhibited a sustained pattern similar to that observed for the total protein release. By comparing the concentration of released VEGF from aerogels to that of liquid platelet lysate, no significant difference was observed, indicating that there is no significant loss of VEGF during the shaping process. This can be attributed to the affinity between VEGF and the fibrin network [46].

### 3.6. Efficacy Assessment of HPL Aerogels Released Products: Metabolic Activity and Cell Migration Analysis 

**Figure 7:**
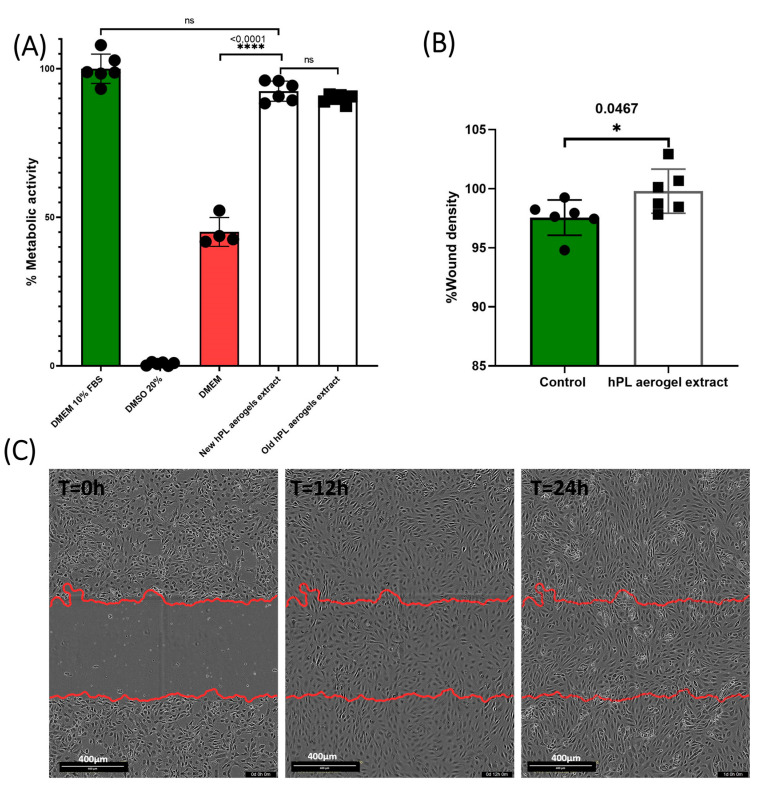
This figure presents the results of the biological evaluation of products released by HPL aerogels. (**A**) The graph presents the metabolic activity of BALB 3T3 cells when exposed to the media conditioned by HPL aerogels, as measured by the MTT assay. Error bars represent standard deviation. (**B**) The graph displays the results of the scratch assay performed on HUVECs. It illustrates the migration capability of HUVECs when exposed to the released products from HPL aerogels. Error bars represent standard deviation. (**C**) The images show the scratch assay at T = 0 h, T = 12 h, and T = 24 h, providing a visual representation of cell migration in response to the released products. The statistical significance of the results was studied using ordinary one-way ANOVA and unpaired t-test with Welch’s correction for *p* < 0.05 (ns > 0.05, * *p* < 0.05, **** *p* < 0.0001). Error bars represent standard deviation (*n* = 6).

The assessment of the effect of the products released by the aerogels (growth factors, cytokines, degradation products, etc.) is carried out using an MTT assay inspired from the recommended indirect method described in ISO 10993-5 standard [47]. The results obtained are presented in Figure 7A. BALB-3T3 cells cultured with the conditioned medium from freshly prepared aerogels (new HPL aerogel extracts) showed a high percentage of metabolic activity (92.45%) with no significant difference to cells treated with the standard culture medium containing 10% FBS (considered as 100%). Contrastingly, cells treated with DMEM alone exhibited a significantly reduced metabolic activity (45.11%) (*p*-value < 0.0001). This finding indicates that the products released from the aerogels retain their functionality and are capable of eliciting a biological response by enhancing the cellular metabolic activity of BALB-3T3 cells. 

To assess the long-term stability of the aerogels, we examined samples that were prepared 24 months prior and stored in the dark at room temperature (old HPL aerogel extracts). These samples demonstrated a nearly equivalent metabolic activity (89.91%) when compared to the freshly prepared aerogels, suggesting that the aerogels maintain their biological efficacy over time when stored appropriately.

These results are consistent with results previously obtained by Andia et al., who showed that platelet derivatives such as such as PRP and HPL in dry form preserve the effectiveness of their bioactive molecules (growth factors, cytokines, etc.) for a duration that can extend to several months [48]. Some even consider lyophilization as an optimal storage method for HPL [49].

Thus, these two drying processes (freeze-drying and drying in supercritical CO_2_) by eliminating water from the aerogels make it possible to avoid the degradation of proteins and other biological compounds present in HPL [50]. Additionally, the removal of water significantly reduces enzymatic oxidation reactions and bacterial growth, contributing to increased stability of the platelet lysate.

Endothelial cell migration is an important mechanism that contributes to tissue formation and is essential for wound healing and tissue regeneration [51].

The results of cellular migration tests conducted on endothelial cells (GFP+-HUVECs) are presented in Figure 7B,C. After 24 h, the cells treated with conditioned media were significantly more confluent at the scratch site compared to cells treated with the control (*p* = 0.0467).

Platelet lysate is rich in factors that promote the migration of endothelial cells, such as PDGF and VEGF. ELISA test confirmed that the aerogels contained and could release VEGF. These results suggest that HPL aerogels contain and release functional pro-angiogenic factors that can promote the migration of endothelial cells.

The results demonstrate that the growth factors originally present in the liquid platelet lysate, or at least a portion of them, are still present and can be released from HPL aerogels and promote the migration of endothelial cells.

### 3.7. Cell Adhesion and Proliferation on Platelet Lysate Aerogels

**Figure 8:**
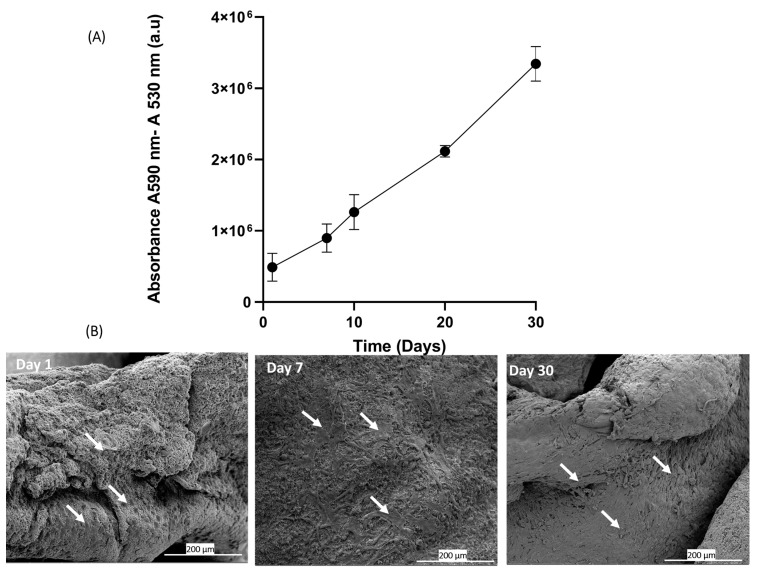
This figure presents the results of cell seeding directly onto HPL aerogels. (**A**) The graph shows the measurement of metabolic activity using the Alamar Blue assay at days 1, 7, 10, 20, and 30. It indicates the viability and metabolic activity of Balb-3T3 cells cultured on HPL aerogels over time (*n* = 4). (**B**) The images display scanning electron microscopy (SEM) images of cells deposited on the HPL aerogels at day 1, day 7, and day 30. The white arrows highlight the cellular morphology.

The in vitro analysis of adhesion and proliferation of BALB-3T3 cells seeded on platelet lysate aerogels was evaluated through the measurement of metabolic activity using Alamar Blue assay. The results graphically presented in Figure 8A clearly demonstrate a significant increase in signal over the course of days, correlating with an increase in metabolic activity and cell number [52]. These results indicate that around 70% of the seeded cells adhere, adapt, and proliferate, suggesting that the three-dimensional fibrin network forming the aerogel maintains its biocompatibility and can be used as a cell culture support. The fibrin network can be considered a supportive substrate for cell adhesion and proliferation [53]. The drying process allows the formation of aerogels that preserve this characteristic, as evidenced by scanning electron microscopy (SEM) images of the seeded cells. On day 1, the cells start to adhere to the network, and over the course of days, an increase in cell density is observed on day 7 and day 30 (Figure 8B).

## 4. Conclusions

Hemoderivative products such as platelet lysate are extensively utilized in tissue engineering due to their rich content of growth factors that facilitate tissue repair and regeneration. However, the use of these substances in hydrogel form is significantly constrained by their limited manipulability and temporal stability. The aerogels developed in this study demonstrate enhanced mechanical properties, offering improved manageability and unprecedented long-term structural and functional stability for blood-derived biomaterials.

For scaffolds and biomaterials to be effective, they must exhibit biocompatibility, a suitable microstructure, mechanical strength, and the ability to support cell residency while releasing bioactive molecules like VEGF effectively. The HPL aerogels, developed through supercritical CO_2_ drying, meet these criteria, positioning them as preferred materials for tissue engineering and as an autologous source of bioactive molecules. Notably, they excel in supporting cell adhesion and proliferation. Furthermore, their application potential in fields such as dental surgery to promote healing and tissue regeneration is significant, particularly due to their growth factor richness, which is conducive to bone and soft tissue formation.

Before contemplating large-scale clinical application, further investigations are essential, starting with the quantification of all growth factors present. While our study confirmed the presence of VEGF post-processing, assessing the concentration of other vital growth factors is crucial. Additionally, despite the recognized efficacy of supercritical CO_2_ as a sterilization method, the sterility of the obtained aerogels must be thoroughly verified, and alternative sterilization methods should be considered if necessary. It is also important to note that the drying process resulted in aerogels that are significantly harder and more manageable than hydrogels, yet their susceptibility to fracturing under high pressure suggests the need to explore additional solutions, such as incorporating natural polymers into the formulation.

## 5. Patents

Platelet lysate foam for cell culture, cell therapy, and tissular regeneration and method for obtaining the same. Publication number: 20230119928.

## Figures and Tables

**Table 1:** Comparison of porosity measurements between HPL hydrogels and aerogels. Porosity measurements obtained through mercury porosimetry for both HPL hydrogels and aerogels, providing quantitative data to support SEM observations (*n* = 3).

	Aerogel Dried Using scCO_2_	Hydrogel Oven Dried at 37 °C
Total porosity between 4 nm and 360 µm (%)	93.9% ± 2%	7.9% ± 4%

## Data Availability

Data from this article are available upon reasonable request.
